# Hydrothermal Corrosion of SiC Coupons Suppressed by Magnetron Sputtered Cr Coatings

**DOI:** 10.1155/2022/4549441

**Published:** 2022-08-31

**Authors:** Shuxin Dai, Zhiming Zou, Renda Wang, Yuhang Li, Bingsheng Li, Fangfang Ge, Peng Li

**Affiliations:** ^1^Institute of Materials, School of Materials Science and Engineering, Shanghai University, Shanghai 200444, China; ^2^Engineering Laboratory of Advanced Energy Materials, Ningbo Institute of Materials Technology and Engineering, Chinese Academy of Sciences, Ningbo 315201, China; ^3^Materials Science and Chemical Engineering, Ningbo University, Ningbo 315211, China; ^4^State Key Laboratory for Environment-Friendly Energy Materials, Southwest University of Science and Technology, Mianyang, 621010 Sichuan, China; ^5^Advanced Energy Science and Technology Guangdong Laboratory, Huizhou 516000, China

## Abstract

SiC-based components are sometimes susceptible to aqueous dissolution in LWR coolant environments. To address this issue, ~10 *μ*m thick Cr coatings was deposited on reaction-bonded silicon carbide (RBSC) plates by magnetron sputtering. Corrosion behavior of Cr-coated SiC and -uncoated SiC coupons was studied by immersing in autoclave (345°C and 16.5 MPa). The weight loss of the Cr coated SiC coupons (3.02% after the 93-days) in the autoclave tests was effectively reduced due to their Cr-coated surfaces, in contrast to the uncoated ones (20.4% after the 78-days). Moreover, microstructural and compositional evolutions were examined by using scanning electron microscopy (SEM), X-ray diffraction (XRD), and Raman spectroscopy. It was revealed that a continuous and dense Cr_2_O_3_ layer formed on the surface after the hydrothermal corrosion, which can suppress the in-diffusion of corrosive medium.

## 1. Introduction

Since the nuclear accident in Japan in 2011, considerable research has been devoted to the development of accident tolerant fuel (ATF) cladding for light-water reactors (LWRs). Nowadays, SiC/SiC_f_ ceramic matrix composites are considered as a potential material for nuclear fuel claddings in USA, Korea, France, and Japan [1–11]. The silicon carbide (SiC) exhibits excellent oxidation resistance under the accident conditions such as in a typical loss of coolant accident (LOCA) scenario [12–16], where a protective SiO_2_ layer can form on the surfaces in the >1000°C steam, preventing breakaway oxidation [17]. However, SiC-based components are prone to hydrothermal corrosion under the supercritical water conditions [7, 9, 18–20], as the SiO_2_ layers on surfaces can dissolve rapidly in the >290°C water [9, 19, 20]. For example, a SiC-based fuel cladding proceeded a recession rate of 0.1 mg/cm^2^-month and thickness loss of ~3.7 mm/year (assuming fully dense cladding), resulting in the dissolution of ~55 kg of SiO_2_ into the primary coolant annually [8]. The main problem we have to address is how to improve the hydrothermal corrosion resistance of SiC at the normal operating conditions.

Recently, it has been proposed that some aqueous corrosion-resistant coatings can be used to mitigate the dissolution of SiC [1, 21, 22]. For example, Oak Ridge National Laboratory successfully deposited adherent CrN, TiN, and Cr coatings onto CVD SiC and SiC/SiC composite coupons and rods via cathodic arc [5]. They have previously compared the hydrothermal corrosion resistance of CVD SiC samples with different coatings, all of which were exposed in an autoclave of 288°C water for 400 hours. The Cr coating exhibited the least amount of mass change [6]. Some initial tests show favorable corrosion resistance of the Cr-based coatings. In addition, The Cr coatings are already applied on the Zr-alloy claddings [4, 23, 24], as their hot water corrosion resistance and high temperature steam oxidation resistance are evaluated as level 5 and level 4 (where 1, worst; …; 5, best) [24], respectively.

This work confirmed that due to the protection of the Cr coatings, the corrosion of reaction-bonded silicon carbide (RBSC) coupons can be effectively suppressed in the supercritical water environment (345°C, 16.5 MPa). Both the Cr-coated SiC coupons and uncoated ones were immersed in the autoclave, followed by the comparison on their evolutions of weight loss, composition, and microstructure. Finally, analysis and discussion were performed on the corrosion resistance mechanism of the Cr-coated SiC in the supercritical water.

## 2. Experimental Details

### 2.1. Coating Preparation

The Cr coatings were deposited on reaction-bonded silicon carbide (RBSC) coupons (density = 3.01 g/cm^3^, 20 mm in diameter, and 2 mm in height, Ningbo Yinzhou seal factory, China) by magnetron sputtering. In the chamber, the Cr target (99.9 at. % in purity) was driven by a radio frequency (RF) power supply (Comdel CV-1000) in parallel to another DC power supply. The deposition parameters are given in Table 1. The thickness of the Cr coatings was about 10 *μ*m.

### 2.2. Coating Characterization

The microstructure of the samples was analyzed by a Bruker D8 Advance X-ray diffraction diffractometer with Cu K*α* radiation at a step of *θ* = 0.02°. The chemical species of the surfaces were probed by Raman spectroscopy on a Renish inVia-reflex system with an excitation laser of 532 nm. The surface and cross-sectional morphologies of the specimens were examined by a Gemini SEM 300 or a FEI Quanta 250™ FEG field emission scanning electron microscope (SEM). The elemental compositions of the specimens were determined by an energy dispersive X-ray spectroscopy (EDS), with an accelerating voltage of 20 keV. The coating-substrate adhesion was evaluated utilizing a scratch tester (CSM Instruments, Switzerland) with a Rockwell C diamond indenter (200 *μ*m in radius) under a load range of 1 − 100 N and a scratch length of 5 mm with a loading rate of 5 N/s. The nanoindentation hardness of the as-deposited coating was tested with MTS Nano Indenter G200 tool with a Berkovich diamond tip (the diameter of 20 nm), and the indentation depth was 1.5 *μ*m. 10 points are randomly tested for each sample, and the average value is finally selected. The corrosion tests were carried out in an autoclave with deionized water at 345°C and 16.5 MPa. Before and after each exposure segment, the samples were weighed on the electronic balance with an accuracy of 0.1 mg for 10 times.

## 3. Results and Discussion

### 3.1. The Microstructure and Mechanical Properties of the As-Deposited Coatings

**Figure 1:**
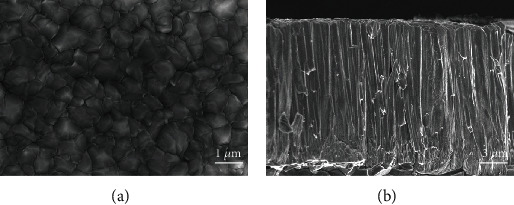
SEM images of the as-deposited Cr coatings (a) surface and (b) cross section.

**Figure 2:**
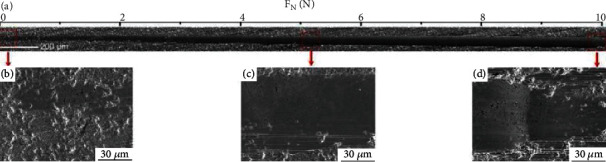
Scratch topography on the as-deposited Cr coating (a) and partially enlarged images of scratch (b–d).

Figure 1 shows the surface and cross-sectional SEM images of the as-deposited Cr coating on the SiC coupon. The coating presents a compact and columnar structure, being the result of competitive growth [25, 26]. The adhesion of the Cr coating on its SiC substrate was evaluated by the scratch method. The morphology of the scratch track is presented in Figure 2. The coating was pressed under the load of 10 N, without any sign of crack or delamination, indicative of good adhesion between the coating and the SiC coupon. The H of the deposited coating obtained by nanoindentation test is~2.022 ± 0.271 GPa.

### 3.2. Corrosion Behavior of the Uncoated SiC in the Supercritical Water

**Figure 3:**
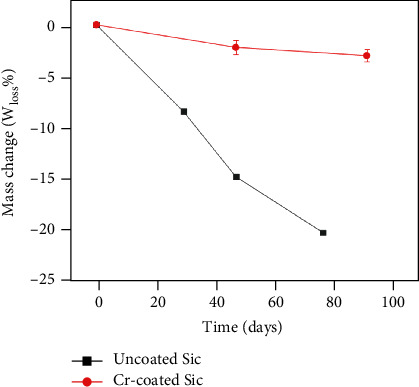
Mass change of the SiC and the Cr-coated SiC specimens after the autoclave immersion.

The uncoated SiC coupons and the Cr-coated ones were simultaneously immersed in an autoclave of supercritical water environment.  Figure 3 lists the weight loss percentages (*W*_Loss_%) of the two kinds of specimens as a function of the immersion times (*t*). A great weight loss occurred in the uncoated coupon during the immersion processes. For example, the *W*_Loss_% values of the uncoated coupon are 8.5%, 14.9%, and 20.4% for *t* = 30, 48, and 78 days, respectively.

**Figure 4:**
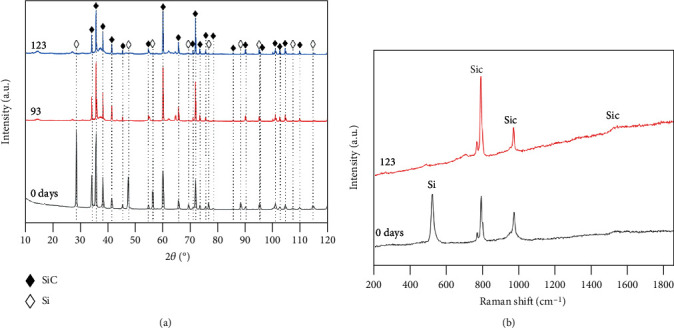
XRD patterns (a) and Raman spectra (b) of the SiC specimens after the immersion tests for various durations.

Figure 4 displays XRD patterns and Raman spectra of the uncoated SiC specimens after the immersion tests for various durations. Both SiC-6H (PDF#29-1131) and Si (PDF#27-1402) phases were mainly observed in Figure 4(a), meaning that some free Si also exists in the SiC coupon. Two peaks of Si at 28.60 cm^−1^ and 47.41 cm^−1^, etc. are evident in the 0-day specimen. Intensities of the two peaks gradually decrease and finally disappear when the immersion time extends to 123 days. By comparison, there is no change for the diffraction peaks of the SiC before and after the immersion process. This observation can suggest that the SiC component did not change during the process, whereas the free Si was preferentially dissolved in the supercritical water. Moreover, the Raman characteristic peaks of SiC are ~788 cm^−1^, ~966 cm^−1^, and~1500 cm^−1^, and the characteristic peak of Si is ~520 cm^−1^ [27] in Figure 4(b). The Raman peak of Si (~520 cm^−1^) decreases gradually with the extension of the immersion time, showing the same change trend as the XRD peaks, which can further confirm that the corrosion of the free Si occurs preferentially.

**Figure 5:**
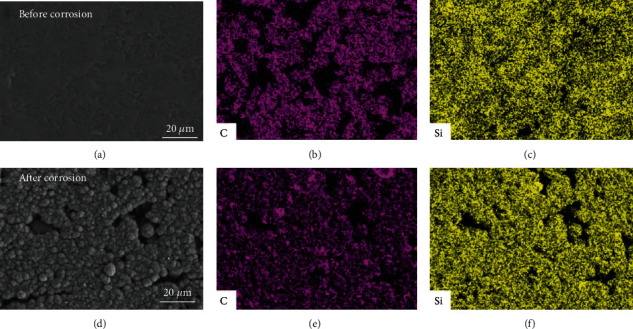
SEM images and EDS mappings of the SiC specimens before (a–c) and after (d–f) the immersion in the autoclave for 93 days.

Figure 5 presents surface morphologies and EDS composition maps of the uncoated SiC specimens before and after the immersion in the autoclave for 93 days. Before the immersion the SiC coupon exhibits intact surface, where the Si-rich regions mostly locate around the SiC grains (Figures 5(a)–5(c)). After the 93-days immersion, many corrosion pits and microcracks appear on the surface of the SiC coupon (Figure 5(d)). Combining with Figures 5(e) and 5(f), it can be inferred that these severely corroded area mainly corresponds to the original Si enriched areas, that is, the free Si is preferentially dissolved in the corrosion process. Kim et al. [18] also found that the weight loss of the RBSC ceramics increases as the content of free Si increases.

The oxidation reaction in the presence of water vapor is considered to progress as follows [28–30]:

(1) $$\begin{array}{c} \text{Si}+2{\text{H}}_{2}\text{O}\left(\text{g}\right)={\text{SiO}}_{2}+2{\text{H}}_{2}\left(\text{g}\right)\;\Delta G=-\frac{373.205\text{ kJ}}{\text{mol}}, \end{array}$$

(2) $$\begin{array}{c} \text{SiC}+2{\text{H}}_{2}\text{O}\left(\text{g}\right)={\text{SiO}}_{2}+{\text{CH}}_{4}\left(\text{g}\right)\;\Delta G=-\frac{327.687\text{ kJ}}{\text{mol}}. \end{array}$$

Generally, the SiO_2_ layer plays a protective role and decreases reaction rates under most conditions. However, the SiO_2_ layer is believed to be unstable in the high-temperature and high-pressure water, and the dissolution of silica into water takes place by [28, 31–33]:

(3) $$\begin{array}{c} {\text{SiO}}_{2}+{\text{nH}}_{2}\text{O}={\text{SiO}}_{2}•{\text{nH}}_{2}\text{O}. \end{array}$$

Hirayama et al. [28] observed that a nonprotective Si (OH)_4_ hydrosilica sol film, instead of an SiO_2_ layer, formed in the high-temperature water. The hydrothermal reaction rate of the Si phase may be faster than that of the SiC phase, which may lead to preferential corrosion of the free Si. To evaluate reaction (1), Opila [29] took a piece of silicon wafer for oxidation at a temperature of 1200°C in a 90 vol% H_2_O/O_2_ mixture. The SiO_2_ scale that generated on the surface remained amorphous and featureless, in contrast to the scale that formed on SiC under identical conditions. The results also proved that the reaction rates of Si and SiC with water are different under the same conditions.

### 3.3. Corrosion Resistance of the Cr-Coated SiC in the Supercritical Water

Figure 3 reveals that the *W*_Loss_% values of the Cr-coated SiC coupons are much lower than those of the uncoated one after the same tests. For example, the *W*_Loss_% of the Cr-coated coupon is only 3.02% for *t* = 93 days, whereas the corresponding value of the uncoated coupon is 20.4% for *t* = 78 days. It demonstrates that the Cr coatings can substantially reduce the corrosion-induced weight loss of the SiC coupons. Subsequent characterization was performed on the Cr-coated SiC coupons that underwent the immersion tests.

**Figure 6:**
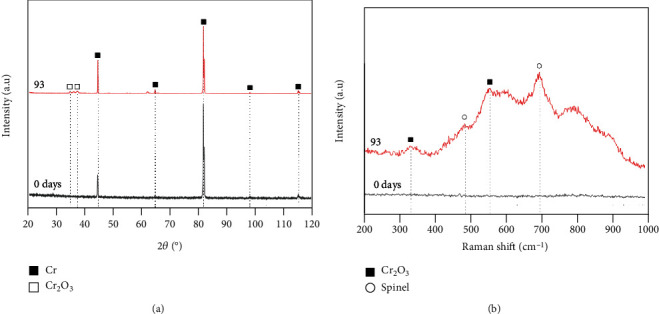
XRD patterns and Raman spectra of the Cr coated SiC specimens after immersion tests for 93 days.

Figure 6 displays XRD patterns and Raman spectra of the Cr-coated SiC specimens before and after the 93-day immersion test. In comparison with the as-deposited specimen, the corroded specimen exhibits similar XRD pattern, except for the appearance of some weak peaks that could be assigned to hexagonal Cr_2_O_3_ (PDF#38-1479) (Figure 6(a)). The Cr (PDF#06-0694) coating remained the original crystal structure during the 93-day immersion in supercritical water. Furthermore, the Raman spectrum in Figure 6(b) can accurately determine the products on the surface, showing the characteristic peaks of Cr_2_O_3_ (~335 cm^−1^ and ~558 cm^−1^) [34] and spinel (~490 cm^−1^ and ~700 cm^−1^) [35, 36]. It indicates that there is indeed a Cr_2_O_3_ layer formed on the coating surface. As for the appearance of spinel (one kind iron-chromium mixed oxides), one potential cause is the incorporation of the impurity Ni or Fe ions into the oxide during its formation. The other is the precipitation of oxides from the coolant, without regard to the oxidation rate of the coatings, each arising from the impurity metals in the autoclave water [37].

**Figure 7:**
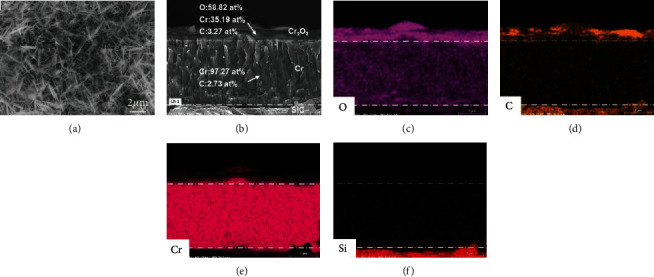
Surface SEM image (a) and cross-sectional SEM image (b) for the corroded Cr-coated SiC specimens that were immersed in autoclave for 93 days. (c–f) show EDS maps of the image (b).

The morphologies and elemental maps of the corroded specimen are displayed in Figure 7. Many whiskers form on the surface, and a 1.0-1.5 *μ*m thick layer of Cr-O scale locates underneath the whiskers layer. The composition analysis shows that the atomic ratios of Cr and O account for ~35.19% and ~58.82% in the Cr-O scale, respectively. The Cr-O scale is covered with some “whiskers,” being common features of Cr after oxidation under a moist atmosphere. Based on the XRD pattern and the Raman spectra, the Cr-O scale mainly composes of Cr_2_O_3_. Guillou et al. [38] also found the thin external Cr-O scale is denser without any pores. Hansel et al. [39] proved that local surface catalysis of H_2_O dissociation at low P_O2_ values causes local acceleration in the oxidation rate and the growth of protruding oxide whiskers. So the corrosion process in the supercritical water can be regarded as a slow oxidation process [40]:

(4) $$\begin{array}{c} 2\text{Cr}\left(\text{s}\right)+3{\text{H}}_{2}\text{O}\left(\text{g}\right)⟶{\text{Cr}}_{2}{\text{O}}_{3}\left(\text{s}\right)+3{\text{H}}_{2}\left(\text{g}\right). \end{array}$$

Additionally, energy spectrum analysis shows that Cr (~97.27 at %) is the main element below the oxide layer, and almost no O composition was detected, which confirms that the reaction (4) occurred on the surface of the Cr coating. The residual Cr coating still maintains the columnar crystal structure and close contact with the Cr_2_O_3_ scale. Our previous work also revealed that very thin Cr_2_O_3_ tissue formed on the outmost surface and along the columnar boundaries during the immersion process [41, 42], which could effectively prevent the inter-diffusion of corrosive medium towards the underneath SiC substrate. Generally speaking, the columnar grain boundary will act as a fast diffusion channel of corrosion medium. Cr_2_O_3_ is preferentially corroded at the columnar grain boundary, and the fast diffusion channel is filled, which may reduce the internal diffusion of oxidant and reduce the corrosion kinetics. At the same time, the layer of dense Cr_2_O_3_ formed on the surface also protects the Cr coating below to prevent further corrosion of the Cr coating. Thus, the Cr coatings can substantially suppress the dissolve corrosion of the SiC coupons in the supercritical water, also presenting potential of application on SiC/SiC_f_ ceramic matrix composite cladding.

## 4. Conclusions

It is confirmed that free Si is preferentially dissolved in hydrothermal corrosion of reaction-bonded silicon carbide. The Cr coatings were successful magnetron sputtered on the RBSC coupons, and Cr coating showed good adhesion to the SiC substrate. The corrosion resistance of the uncoated SiC coupons and the Cr-coated SiC coupons was simultaneously evaluated in the 345°C and 16.5 MPa autoclave. The weight loss percentage of the coated SiC coupons was only 3.02% after the 93-day immersion in the autoclave, in contrast to that the weight loss percentage of the uncoated SiC coupons reach up to 20.4% after the 78-day immersion. The results showed that the corrosion resistance of SiC can be greatly improved by plating Cr coating. Meanwhile, it demonstrates that the coatings can prevent the SiC coupons from the corrosion of supercritical water, due to a continuous and dense Cr_2_O_3_ layer formed on the surface. The work would be helpful for developing new generation ATF coatings of SiC claddings.

## Figures and Tables

**Table 1:** Deposition conditions of Cr coatings.

Target	Cr (99.9%)
Base pressure	~1.0∗10^−5^ Pa
Ar pressure	0.7 Pa
Ar gas flow rate	32 SCCM
Revolution speed	12 rmp
Cr target power (MF-DC)	200 W
Radio frequency (RF)	250 W
Substrate bias voltage (DC)	-5 V
Deposition temperature	400 K

## Data Availability

The data used to support the findings of this study are included within the article.

## References

[1] Ang C. K., Terrani K. A., Burns J., Katoh Y. (2016). Examination of hybrid metal coatings for mitigation of fission product release and corrosion protection of LWR SiC/SiC. *ORNL/TM-2016/332*.

[2] Braun J., Guéneau C., Alpettaz T. (2017). Chemical compatibility between UO_2_ fuel and SiC cladding for LWRs. Application to ATF (Accident-Tolerant Fuels). *Journal of Nuclear Materials*.

[3] Kim D., Lee H. J., Jang C., Lee H. G., Park J. Y., Kim W. J. (2017). Influence of microstructure on hydrothermal corrosion of chemically vapor processed SiC composite tubes. *Journal of Nuclear Materials*.

[4] Kim H. G., Yang J. H., Kim W. J., Koo Y. H. (2016). Development status of accident-tolerant fuel for light water reactors in Korea. *Nuclear Engineering and Technology*.

[5] Mouche P. A., Ang C., Koyanagi T., Doyle P., Katoh Y. (2019). Characterization of PVD Cr, CrN, and TiN coatings on SiC. *Journal of Nuclear Materials*.

[6] Raiman S. S., Ang C., Doyle P., Terrani K. A. Hydrothermal Corrosion of SiC materials for accident tolerant fuel cladding with and without mitigation coatings.

[7] Raiman S. S., Doyle P., Ang C., Katoh Y., Terrani K. A. (2019). Hydrothermal corrosion of coatings on silicon carbide in boiling water reactor conditions. *Corrosion*.

[8] Terrani K. A. (2018). Accident tolerant fuel cladding development: promise, status, and challenges. *Journal of Nuclear Materials*.

[9] Terrani K. A., Yang Y., Kim Y. J., Rebak R., Meyer H. M., Gerczak T. J. (2015). Hydrothermal corrosion of SiC in LWR coolant environments in the absence of irradiation. *Journal of Nuclear Materials*.

[10] Zinkle S. J., Terrani K. A., Gehin J. C., Ott L. J., Snead L. L. (2014). Accident tolerant fuels for LWRs: a perspective. *Journal of Nuclear Materials*.

[11] Konishi H., Idris M. I., Imai M., Yoshida K., Yano T. (2013). Neutron irradiation effects of oxide sintering additives for SiCf/SiC composites. *Energy Procedia*.

[12] Azevedo C. R. F. (2011). Selection of fuel cladding material for nuclear fission reactors. *Engineering Failure Analysis*.

[13] Pham H. V., Nagae Y., Kurata M., Bottomley D., Furumoto K. (2020). Oxidation kinetics of silicon carbide in steam at temperature range of 1400 to 2020 °C studied by laser heating. *Journal of Nuclear Materials*.

[14] Pint B. A., Terrani K. A., Brady M. P., Cheng T., Keiser J. R. (2013). High temperature oxidation of fuel cladding candidate materials in steam- hydrogen environments. *Journal of Nuclear Materials*.

[15] Pint B. A., Terrani K. A., Yamamoto Y., Snead L. L. (2015). Material selection for accident tolerant fuel cladding. *Metallurgical and Materials Transactions E*.

[16] Raiman S. S., Field K. G., Rebak R. B., Yamamoto Y., Terrani K. A. (2020). Hydrothermal corrosion of 2nd generation FeCrAl alloys for accident tolerant fuel cladding. *Journal of Nuclear Materials*.

[17] Doyle P. J., Koyanagi T., Ang C. (2020). Evaluation of the effects of neutron irradiation on first-generation corrosion mitigation coatings on SiC for accident-tolerant fuel cladding. *Journal of Nuclear Materials*.

[18] Kim W. J., Hwang H. S., Park J. Y. (2002). Corrosion behavior of reaction-bonded silicon carbide ceramics in high-temperature water. *Journal of Materials Science Letters*.

[19] Kim W. J., Hwang H. S., Park J. Y., Ryu W. S. (2003). Corrosion behaviors of sintered and chemically vapor deposited silicon carbide ceramics in water at 360 360 °C. *Journal of Materials Science Letters*.

[20] Park J. Y., Kim I. H., Jung Y. I., Kim H. G., Park D. J., Kim W. J. (2013). Long-term corrosion behavior of CVD SiC in 360 °C water and 400 °C steam. *Journal of Nuclear Materials*.

[21] Ang C., Katoh Y., Kemery C., Kiggans J., Terrani K. (2016). Chromium-based mitigation coatings on SiC materials for fuel cladding. *2016 Transactions of the American Nuclear Society Annual Meeting, ANS*.

[22] Ishibashi R., Ishida K., Kondo T., Watanabe Y. (2021). Corrosion-resistant metallic coating on silicon carbide for use in high- temperature water. *Journal of Nuclear Materials*.

[23] Kim H. G., Kim I. H., Jung Y. I., Park D. J., Park J. Y., Koo Y. H. (2015). Adhesion property and high-temperature oxidation behavior of Cr-coated Zircaloy-4 cladding tube prepared by 3D laser coating. *Journal of Nuclear Materials*.

[24] Tang C., Stueber M., Seifert H. J., Steinbrueck M. (2017). Protective coatings on zirconium-based alloys as accident-tolerant fuel (ATF) claddings. *Corrosion Reviews*.

[25] Petrov I., Barna P. B., Hultman L., Greene J. E. (2003). Microstructural evolution during film growth. *Journal of Vacuum Science & Technology A*.

[26] Adamik M., Barna P. B., Tomov I. (1998). Columnar structures in polycrystalline thin films developed by competitive growth. *Thin Solid Films*.

[27] Krautwasser P., Begun G. M., Angelini P. (1983). Raman spectral characterization of silicon carbide nuclear fuel coatings. *Journal of the American Ceramic Society*.

[28] Hirayama H., Kawakubo T., Goto A., Kaneko T. (1989). Corrosion behavior of silicon carbide in 290°C water. *Journal of the American Ceramic Society*.

[29] Opila E. J. (1999). Variation of the oxidation rate of silicon carbide with water-vapor pressure. *Journal of the American Ceramic Society*.

[30] Barin I. (1995). *Thermochemical Data of Pure Substances*.

[31] Gogotsi Y., Yoshimura M. (1995). Degradation of SiC (Tyranno) fibres in high-temperature, high-pressure water. *Journal of Materials Science Letters*.

[32] Hackley V. A., Paik U., Kim B. H., Malghan S. G. (1997). Aqueous processing of sintered reaction-bonded silicon nitride: I, dispersion properties of silicon powder. *Journal of the American Ceramic Society*.

[33] Jacobson N. S., Gototsi Y. G., Yoshimura M. (1995). Thermodynamic and experimental study of carbon formation on carbides under hydrothermal conditions. *Journal of Materials Chemistry*.

[34] Mougin J., Rosman N., Lucazeau G., Galerie A. (2001). In situ Raman monitoring of chromium oxide scale growth for stress determination. *Journal of Raman Spectroscopy*.

[35] Baraton M. I., Busca G., Prieto M. C., Ricchiardi G., Escribano V. S. (1994). On the vibrational spectra and structure of FeCrO_3_ and of the ilmenite-type compounds CoTiO_3_ and NiTiO_3_. *Journal of Solid State Chemistry*.

[36] D'Ippolito V., Andreozzi G. B., Bersani D., Lottici P. P. (2015). Raman fingerprint of chromate, aluminate and ferrite spinels. *Journal of Raman Spectroscopy*.

[37] Doyle P. J., Ang C., Snead L., Katoh Y., Terrani K., Raiman S. S. (2021). Hydrothermal corrosion of first-generation dual-purpose coatings on silicon carbide for accident-tolerant fuel cladding. *Journal of Nuclear Materials*.

[38] Guillou S., Cabet C., Desgranges C., Marchetti L., Wouters Y. (2011). Influence of hydrogen and water vapour on the kinetics of chromium oxide growth at high temperature. *Oxidation of Metals*.

[39] Hansel M., Quadakkers W. J., Young D. J. (2003). Role of water vapor in chromia-scale growth at low oxygen partial pressure. *Oxidation of Metals*.

[40] Lee Y., Koyanagi T., Pint B. A., Kato Y. (2019). *High temperature steam oxidation of Cr-coated SiCf/SiC composite for LWR cladding applications*.

[41] Liu H., Feng Y., Yao Y. (2021). Effect of the 345 °C and 16.5 MPa autoclave corrosion on the oxidation behavior of Cr-coated zirconium claddings in the high-temperature steam. *Corrosion Science*.

[42] Shi X., Li B., Liu H. (2021). The corrosion resistance mechanisms of the cr-coated SiC in molten Na_2_SO_4_ salt: strengthened boundaries and protective scales. *Corrosion Science*.

